# High-Resolution Magic-Angle-Spinning NMR in Revealing Hepatoblastoma Hallmarks

**DOI:** 10.3390/biomedicines10123091

**Published:** 2022-12-01

**Authors:** Ljubica Tasic, Nataša Avramović, Milka Jadranin, Melissa Quintero, Danijela Stanisic, Lucas G. Martins, Tássia Brena Barroso Carneiro Costa, Estela Novak, Vicente Odone, Maria Rivas, Talita Aguiar, Dirce Maria Carraro, Isabela Werneck da Cunha, Cecilia Maria Lima da Costa, Mariana Maschietto, Ana Krepischi

**Affiliations:** 1 Chemical Biology Laboratory, Department of Organic Chemistry, Institute of Chemistry, University of Campinas (UNICAMP), Campinas 13083-970, SP, Brazil; 2 Institute of Medical Chemistry, Faculty of Medicine, University of Belgrade, 11000 Belgrade, Serbia; 3 Institute of Chemistry, Technology, and Metallurgy, Department of Chemistry, University of Belgrade, 11000 Belgrade, Serbia; 4 Pediatric Cancer Institute (ITACI), Pediatric Department, Sao Paulo University Medical School, Sao Paulo 05403-901, SP, Brazil; 5 Human Genome and Stem Cell Research Center, Department of Genetics and Evolutionary Biology, Institute of Biosciences, University of Sao Paulo, Sao Paulo 05508-090, SP, Brazil; 6 Columbia University Irving Medical Center, New York, NY 10032, USA; 7 International Center for Research, A. C. Camargo Cancer Center, Sao Paulo 01509-010, SP, Brazil; 8 Pathology Department, Rede D’OR-São Luiz, Sao Paulo 04321-120, SP, Brazil; 9 Department of Pediatric Oncology, A. C. Camargo Cancer Center, Sao Paulo 01509-010, SP, Brazil; 10 National Laboratory of Biosciences (LNBio), National Center for Research in Energy and Materials (CNPEM), Campinas 13083-100, SP, Brazil

**Keywords:** hepatoblastoma, liver metabolome, cancer NMR-metabolomics

## Abstract

Cancer is one of the leading causes of death in children and adolescents worldwide; among the types of liver cancer, hepatoblastoma (HBL) is the most common in childhood. Although it affects only two to three individuals in a million, it is mostly asymptomatic at diagnosis, so by the time it is detected it has already advanced. There are specific recommendations regarding HBL treatment, and ongoing studies to stratify the risks of HBL, understand the pathology, and predict prognostics and survival rates. Although magnetic resonance imaging spectroscopy is frequently used in diagnostics of HBL, high-resolution magic-angle-spinning (HR-MAS) NMR spectroscopy of HBL tissues is scarce. Using this technique, we studied the alterations among tissue metabolites of ex vivo samples from (a) HBL and non-cancer liver tissues (NCL), (b) HBL and adjacent non-tumor samples, and (c) two regions of the same HBL samples, one more centralized and the other at the edge of the tumor. It was possible to identify metabolites in HBL, then metabolites from the HBL center and the border samples, and link them to altered metabolisms in tumor tissues, highlighting their potential as biochemical markers. Metabolites closely related to liver metabolisms such as some phospholipids, triacylglycerides, fatty acids, glucose, and amino acids showed differences between the tissues.

## 1. Introduction

Metabolomics provides a complete set of qualitative and quantitative analyses of diverse mixtures of low-molecular-weight compounds in cells, tissues, and body fluids and is mostly assessed by three analytic techniques: nuclear magnetic resonance spectroscopy (NMR), gas chromatography coupled to mass spectrometry (GC-MS), and liquid chromatography coupled to mass spectrometry (LC-MS) [1,2,3,4,5]. Intact tissue samples can be analyzed by high-resolution magic-angle-spinning nuclear magnetic resonance spectroscopy (HR-MAS-NMR) [3,4,5], which uses samples spinning at high speeds at an angle of 54.74° relative to the permanent magnetic field [3,6,7]. HR-MAS-NMR spectroscopy has a promising future to explore metabolites and their involvement in biochemical pathways due to its great advantages. These include simple preparation of samples, high reproducibility, determination of either known or unknown molecule structures, and lastly, the possibility to analyze in vivo and ex vivo samples, which is especially important for clinical research [3,4,5,6,7,8,9]. Nevertheless, NMR spectroscopy has the disadvantage of lower sensitivity, and a smaller number of compounds can be determined than applying MS.

HR-MAS-NMR is applied in metabolomics studies aiming to determine the current pathophysiological state of organisms/tissues/organs and define disease outcomes [10]. Metabolomics has an important role in the pediatric oncology field of research, contributing to evidence of alterations in the type and concentration of metabolites and delineating specific metabolic profiles that are important for cancer diagnosis and improvement of clinical treatment [6,7,10,11]. Due to the high heterogeneity of cancer, one of the important challenges of NMR spectroscopy is that numerous metabolites are being analyzed and resolved [3,6,7]. Although cancer is clonal, the neoplastic process is dynamic and tumors are cellularly heterogeneous and can present metastatic phenotypes [12]. The high mutagenic potential of heterogeneous cancer cells provides significant metabolic changes that can distinguish malignant from non-malignant cells of the same tumor [12].

Hepatoblastoma (HBL) is the most prevalent liver cancer in children representing around 1% of all pediatric tumors [13,14]. HBL commonly carries driver mutations in the oncogene *CTNNB1* which activates the Wnt/β-catenin pathway [15,16,17,18,19]. The activation of the Wnt/β-catenin pathway leads to liver tumorigenesis and the alteration of cancer cell metabolism [19]. A common clinical feature of HBL is the elevated level of α-fetoprotein (AFP) in serum [20,21], and abdominal mass and distention. As far as we know, no data have been yet reported about HR-MAS ^1^H-NMR of HBL tissue samples except ^1^H-NMR metabolomics analysis in vitro of hepatoblastoma cell lines (HepG2) treated with aflatoxin AFM1 and compared with untreated HepG2 cells [22]. The metabolomics study of HBL was explored by LC-MS to determine the differences in metabolite profiles between HBL cells with overexpression and normal expression of sodium-taurocholate co-transporting polypeptide NTCP (*SLC10A1*) [2]. It was established that adenosine concentration was lower in HBL cells with overexpressed NTCP (*SLC10A1*), leading to the hypothesis that NTCP (*SLC10A1*) can diminish adenosine metabolism.

This study explored for the first time the alterations among tissue metabolites of intact samples (ex vivo) from HBL compared with non-cancer liver tissues (NCL) by HR-MAS ^1^H-NMR. We also compared differences in tissue metabolites between two regions of the same HBL samples, one more centralized and the other at the border of the tumor. It was possible to characterize metabolites that differed among samples from the same patients, and center and border samples and their link to altered metabolisms, highlighting their potential as biochemical markers. Therefore, this study may open the opportunity to monitor HBL for personalized diagnostics and treatment.

## 2. Materials and Methods

### 2.1. Samples

Thirty fresh frozen tissue samples (1–30) were used in this study and provided by the Biobank of two cancer hospitals in Sao Paulo, Brazil—A. C. Camargo Cancer Center (ACCCC), and the Pediatric Cancer Institute (ITACI). Fifteen of the analyzed tissue samples were taken as biopsy specimens from young patients diagnosed with HBL, of which twelve samples were obtained in pairs from the center region and the border of HBL tumors. A further three samples were taken from three patients, but it was unknown from which part of the HBL tissues. Another fifteen samples were taken as non-cancer liver (NCL) as control samples, of which nine samples (16–24) were obtained from the healthy part of the liver from patients diagnosed with HBL (adjacent non-HBL samples), and six samples (25–30) were taken from children with healthy livers.

All HBL patients were treated with pre-chemotherapy. Liver samples were collected and stored frozen at −80 °C until NMR analysis. Patients were followed by clinical examination, imaging tests, and measurement of α-fetoprotein for a minimum of 18 months. Patients were stratified into either high- (8/15), intermediate- (5/15 cases), or low-risk (2/15) groups. Eleven (out of fifteen) patients were male. The Research Ethics Committees approved this project under registration number 1987/14 (retrospective study) and informed consent was obtained from the patients’ legal guardians.

To carry out the analyses, a small portion of the tissues (around 10 mg) was defrosted (ice bath at 4 °C) and cut with the help of a bistoury in sterile conditions. A drop of deuterium oxide was then added to the tissue to facilitate its handling. With the aid of a pipette tip, the piece of tissue was inserted into a 12 μL zirconia rotor. Over the tissue, another 10 μL of deuterium oxide was added. The insert was placed into the rotor allowing excess deuterium oxide to exit through its orifice. The rotor was closed and inserted into the spectrometer for measurements.

### 2.2. Metabolomics by NMR Spectroscopy

HR-MAS ^1^H-NMR spectroscopy with a magic-angle spinning frequency of 3.5 kHz and 298 K was used to analyze fifteen HBL and fifteen NCL tissue samples. The ^1^H-NMR spectra were recorded using a Bruker Avance spectrometer (Bruker BioSpin, Germany) operating at 400 MHz and equipped with the triple nuclei 4 mm probe for HR-MAS. One-dimensional water-suppressed ^1^H-NMR spectra were performed with the nuclear Overhauser effect spectroscopy (NOESY1D) pulse sequence and 256 repeats, and the ^1^H-NMR T_2_-edited spectra were recorded using the CPMG (Carr–Purcell–Melboom–Gill) pulse sequence with 128 repetitions. Two-dimensional total correlation spectroscopy (TOCSY) experiments were performed with 256 scans of randomly selected samples from two tissue groups. Chemometrics analysis was carried out through the open-access platform MetaboAnalyst (www.metaboanalyst.ca, accessed on 20 August 2022) At the same time, the metabolites were identified using 1D and 2D spectral data and databases, such as the Human Metabolome Database (HMDB) and BioMagResBank (BMRB) [23,24].

### 2.3. Statistical Analysis of NMR Data

Using the spectral differences that were identified by analyzing the 1H-NMR T2-edited spectra, the matrices were constructed from 30 spectra (15 for the HBL group and 15 for the NLC group) with 1764 variables from two spectral regions, δ 0.50–4.50 and 5.50–9.00. The first principal component analysis (PCA) was performed on all 30 samples with the objective to investigate the inherent groupings within samples and evaluate eventual outliers. Two additional principal component analyses (PCA) were performed, one with the spectra of the paired samples of HBL and NCL tissues from the same patients (10 spectra in total, 5 for HBL tissues and 5 for NCL tissues), and the other with the spectra of the paired samples of HBL tissues taken from the center and border of the tumor (12 spectra in total, 6 from the center and 6 from the edge of HBL tissue). Partial least squares-discriminant analysis (PLS-DA) was applied to the NMR data to find metabolite dissimilarities between the groups. Following the built PLS-DA models, variable importance in projection (VIP) scores were evaluated to define the most different chemical shifts among the studied samples.

## 3. Results

A total of 30 samples (15 HBL and 15 NCL) were analyzed by HR-MAS ^1^H-NMR to determine the intact tissue metabolites and metabolomic differences among HBLs and NCLs, as well as possible differences in metabolomic profiles of HBL taken from two regions of the same tumor. Two types of 1D NMR experiments by HR-MAS were recorded for metabolomic fingerprinting of the tissues, nuclear Overhauser enhancement spectroscopy (NOESY1D), and ^1^H-NMR T_2_-edited (CPMG) to analyze low-molecular-mass metabolites. Data analysis showed that the most important spectral differences among HBL and NCL tissue samples were in the aliphatic region (δ 0.50–4.50), followed by δ 5.50–9.00 aromatic region of ^1^H-NMR CPMG spectra. Therefore, these spectral regions were used to construct the metabolomics datasets.

**Figure 1:**
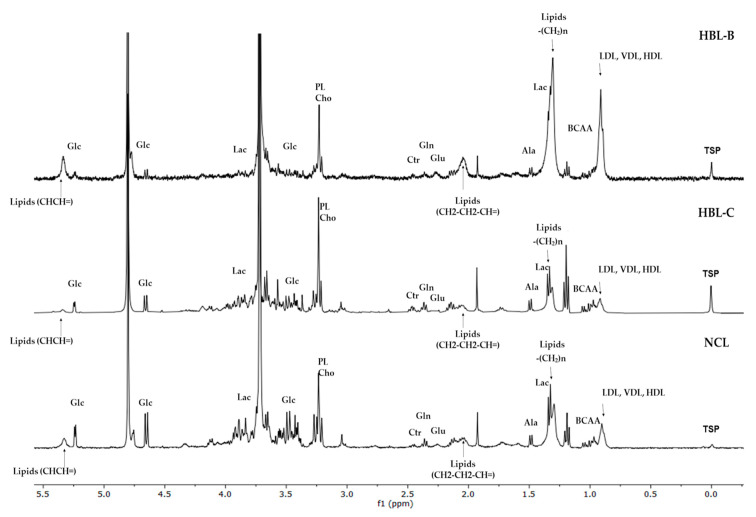
HR-MAS ^1^H-NMR spectra of liver tissue samples, from the bottom to the top: non-cancer liver (NCL) sample, hepatoblastoma sample (HBL-C) taken from the center of the tumor, and hepatoblastoma sample taken from the border of the tumor (HBL-B) in 0.0-5.5 ppm. Identification of the tissue metabolites is given in Table 1.

The spectral differences in aliphatic regions were observed for 30 analyzed samples (15 HBLs and 15 NCLs) and 10 paired HBL/NCL samples as illustrated in Figure 1.

**Figure 2:**
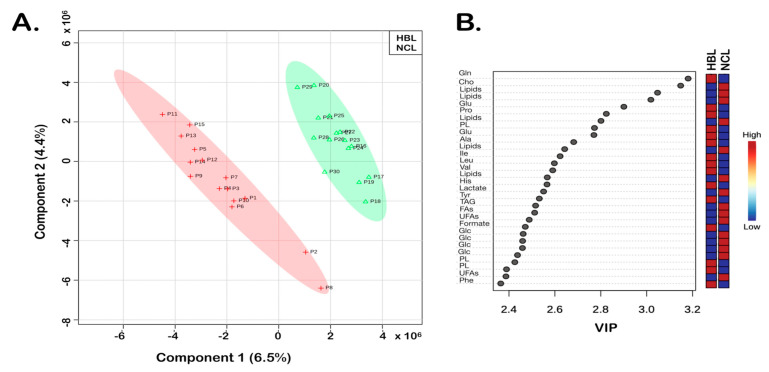
HBL vs. NCL. PLS-DA of the HR-MAS ^1^H-NMR CPMG data: (**A**) 2D score plot shows 15 HBL and 15 NCL samples; statistical parameters were as follows: accuracy (0.68), R^2^ (0.77), and Q^2^ (0.39). (**B**) Variable importance in projection (VIP) scores greater than 2.35 were assigned to important metabolites 1–16, as summarized in Table 1, which are discriminatory for HBL vs. NCL in the PLS-DA model. HBL tissue samples are shown with red crosses and the non-cancer liver tissue samples (NCL) are shown in green triangles.

**Figure 3:**
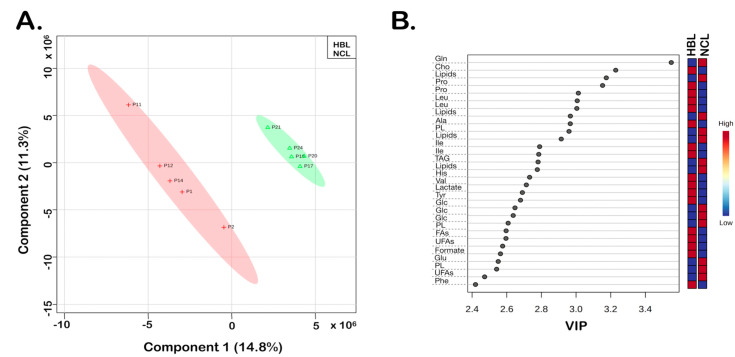
Paired HBL vs. NCL samples, PLS-DA of the HR-MAS ^1^H-NMR CPMG data: (**A**) 2D score plot showing five HBL and five NCL samples; statistical parameters were as follows: accuracy (0.48), R^2^ (0.57), and Q^2^ (0.03). (**B**) Variable importance in projection (VIP) scores greater than 2.4 were assigned to important metabolites 1–16 (Table 1). HBL tissue samples are shown in red crosses and the non-cancer liver tissue samples (NCL) are shown with green triangles.

**Figure 4:**
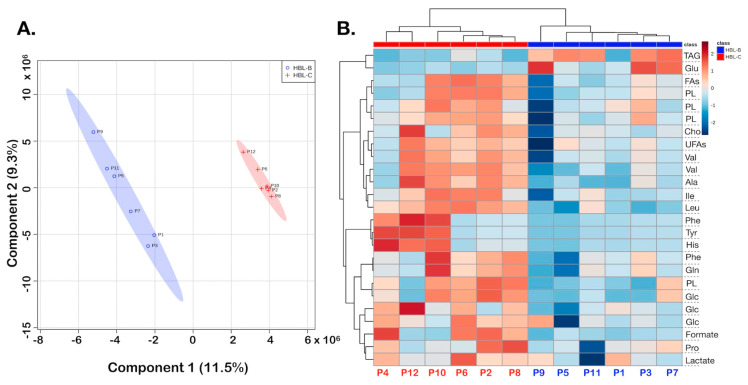
HBL border vs. center, PLS-DA of the HR-MAS ^1^H-NMR CPMG data in the analysis of the cancer samples from border and center, HBL-B and HBL-C, respectively: (**A**) 2D score plot; statistical parameters were as follows: accuracy (0.71), R^2^ (0.87), and Q^2^ (0.23). (**B**) Heatmap showing discriminatory metabolites for the cancer samples. HBL-B tissue samples are shown in blue and HBL-C are shown in red.

At the same time, the spectral features were significantly different in 12 paired HBL samples taken from the center and the border of the tumor. Additionally, the observed differences were revealed by the results of the executed chemometrics analysis (Figure 2, Figure 3 and Figure 4). Using the PLS-DA as a supervised method, the sample plots (Figure 2A, Figure 3A and Figure 4A) showed the analyzed sample group membership. The statistical parameters for group discriminations were satisfactory, regarding accuracies and R^2^, although the Q^2^ values showed somewhat overfitting of the results, principally because of the low number of samples. Nevertheless, clear discriminations between the tumor HBL samples and the NCL were achieved (Figure 2A and Figure 3A). Figure 2B and Figure 3B illustrate the variable importance in the projection (VIP) scores that were computed to determine discriminatory metabolites from the assigned NMR data. Both results of the PLS-DA revealed VIP values greater than 2.35 that belong to identified metabolites, whose abbreviations are given in Table 1. Furthermore, the color of the boxes showed increased (red) and decreased (blue) metabolites, respectively (Figure 2B and Figure 3B).

HBL and NCL samples showed differences in 16 metabolites (Table 1) with the highest contributions identified by VIP values higher than 2.35 (Figure 2B). Five out of sixteen metabolites identified by 1D and 2D NMR data (TOCSY) showed reduced and eleven increased concentrations of HBL compared with NCL (Figure 2B). Among the five metabolites with decreased concentrations (boxes in blue, Figure 2B and Figure 3B), were lactate, glucose (Glc), and triacylglycerides (TAGs) with saturated (FAs) and unsaturated (UFAs) fatty acids used as energy reserve sources. The greatest differences observed for metabolites with increased concentrations (boxes in red, Figure 2B and Figure 3B) were in glutamine (Gln), glutamate (Glu), formate, some aromatic (phenylalanine—Phe, and tyrosine—Tyr), and aliphatic amino acids, especially alanine (Ala), and phospholipids (PL), which are structural lipids and the main components of cell membranes.

According to the PLS-DA results of the paired tumor samples, and the heatmap (Figure 4), border and center samples showed variations in the relative metabolite levels measured in NMR analyses, which are illustrated in color change intensities of increased and decreased metabolites given in red and blue, respectively (Figure 4B). Columns represent samples, with red labels showing HBL-center samples. Rows represent distinct chemical shifts assigned to indicated metabolites (Table 1). For example, it can be seen that unsaturated fatty acid (UFA) levels show opposite trends in the center and border of the cancer samples, and the red color prevails in the center tumor samples. At the same time, TAG levels (first row, Figure 4B) were lower in the tumor center.

The metabolites that contributed to distinguishing both regions of HBL tumors (Figure 4) are triacylglycerides (TAGs), and glutamate (Glu) with decreased concentrations in the center of HBL, whereas concentrations of aliphatic amino acids (alanine—Ala, valine—Val, leucine—Leu, and isoleucine—Ile), lactate, glucose, and phospholipids were increased compared with tissue samples from the border of HBL (Figure 4B). It should be noted that according to data from the most important variables with VIP greater than 2.4, triacylglycerides with especially unsaturated fatty acids showed great differences between HBL tissues taken from the center and border of the tumors.

## 4. Discussion

Due to the rarity of HBL, the studied cohort of samples is significant and especially valuable because the paired HBL and NCL samples had been through the same medical treatment. Differences in metabolite concentrations in HBL compared with NCL tissue samples indicate altered metabolomic pathways and potential diagnostic biomarkers which might be useful as possible targets in clinical treatments. To date, *α*-fetoprotein (AFP) is the only clinical biomarker of HBLs [25]. AFM1 is a hydroxylated metabolite of aflatoxin B1 (AFB1), known as a human carcinogen of the Group 1 type. It was found that AFM1 affects the reprogramming of lipidic, glycolytic, and amino acid metabolism and causes inhibition of hepatoblastoma HepG2 cells. Reported data revealed that HepG2 untreated cells compared with treated cells with AFM1 showed increased concentrations of formate and decreased concentrations of acyl groups of fatty acids, cholesterol, pyruvate/lactate, glycine, choline, phosphorylcholine (PC), glycerophosphorylcholine (GPC), branched-chain amino acids (BCAA), and glutamate [22]. Our results are in agreement with this in terms of decreased concentrations of pyruvate/lactate, glucose, and triglyceride lipids and increased concentrations of formate. Conversely, there is disagreement in terms of increasing concentrations of glutamine/glutamate, aliphatic (alanine) amino acids, and phospholipids compared with reported data. Recently reported studies confirmed the diminished effect of taurocholate co-transporting polypeptide NTCP, encoded by *SLC10A1*, in hepatoblastoma HepG2 cells, and its effect was studied by LC-MS exploring the metabolomic differences between HepG2 with overexpression and normal expression of NTCP (*SLC10A1*) [2]. Our results, compared with data on LC-MS-based metabolomics of HepG2 with normal NTCP expression, show good congruence with increased concentrations of phospholipids and aliphatic amino acid leucine, and decreased concentrations of triglycerides (lipids) and lactate/pyruvate. Additionally, our data show opposite results because of the aromatic amino acids phenylalanine and tyrosine [2].

Cancer cells adapt to the nutrient-deficient microenvironment they need as a source of energy by altering glycolysis, lipids, and amino acids’ metabolisms, in order to survive, proliferate and promote tumorigenesis [26,27,28]. The observed reduction in triglyceride concentrations in all HBL samples indicates that cancer cells use this class of lipids as an alternative source of energy necessary for tumor progression, and an increase in phospholipid concentrations indicates that their synthesis is necessary for structural components of membranes, both cytoplasmic and organelles [27,28,29,30,31]. Depleted glucose and pyruvate concentrations in all HBL versus healthy NCL tissue samples demonstrated enhanced glycolysis that is typical for the so-called Warburg effect [23], wherein the energy metabolism of cancer cells is switched from oxidative phosphorylation in mitochondria to aerobic glycolysis. In fact, glucose is transformed to pyruvate in glycolytic metabolism, and pyruvate is further converted to lactate by the enzyme lactate dehydrogenase (LDH) instead of continuing to participate in the tricarboxylic acid cycle (TCC). We recently reported that the reduction of lipids in HBLs is positively correlated with nicotinamide N-methyltransferase (*NNMT*) downregulation, proposing that these cancer cells are consuming triglycerides as their requirement for energy in tumorigenesis [32]. Reduction of triglycerides and increase of phospholipids were also observed in HBL samples taken from the center versus HBL samples taken from the border of tumors.

The amino acid glutamine (Gln) also has a crucial role in energy metabolism, in addition to proteins, and nucleotide synthesis and is therefore responsible for cell viability and cancer growth [33]. The expression of the *GLUL* which encodes the enzyme glutamine synthetase (GLUL), and is involved in Gln synthesis, is regulated by the Wnt/β-catenin pathway. It was reported that *CTNNB1* mutations, which are commonly detected in HBLs, lead to the reduction of β-catenin degradation and *GLUL* overexpression [10,34], which follows our results; namely, there is an increased Gln/Glu concentration in HBL compared with NCL samples.

We also found significantly altered metabolite concentrations comparing HBL center and border samples from the tumors. The essential difference was the opposite alteration of concentrations of pyruvate/lactate, glucose, glutamine, and glutamate. Concentrations of pyruvate/lactate and glucose were increased whereas glutamine and glutamate concentrations were decreased in HBL samples taken from the center compared with the samples from the border of the tumor.

Indeed, the heterogeneity of HBLs is reflected in the existence of different cell types including pure fetal type, embryonic type, mixed type, and small-cell undifferentiated type [35]. Different HBL subtypes indicate diverse gene expression patterns as well as unique metabolic routes. Crippa et al. showed that the embryonal type of HBL cells has enhanced glycolysis with high activity of lactate dehydrogenase (LDH) and glycolytic enzyme GLUT3, whereas the fetal type of HBL cells has enhanced glutamine metabolism with glucose-6-phosphatase kinase (G6PC) upregulation [36]. Although reported data indicated that glutamine reduction is typical for HBL fetal type [36], it was also detected in the embryonal HBL cell type [34].

Regarding the high heterogeneity of HBL samples (1–12), our findings suggested that HBL samples taken from the center of cancer indicate enhanced glutamine metabolism that corresponds to the fetal cell type, whilst HBL samples taken from the border demonstrate enhanced glycolysis that corresponds to the embryonal undifferentiated cell-type. Therefore, our results reinforce that intratumoral heterogeneity can modify the findings and that it is important to document and accurately describe the location of the studied cancer tissue.

The low mutagenic potential of our analyzed HBL tissue samples and a number of cases prevent us from comparing different HBL types. Another limitation is that all samples were taken from patients that underwent pre-chemotherapy, and treatments can also influence the validation of our findings.

## 5. Conclusions

HR-MAS ^1^H-NMR-based metabolomics study of HBL compared with NCL tissue samples of the same patients revealed different metabolic profiles and altered lipid metabolism, glycolysis, and glutaminolysis in this pediatric liver cancer. Decreased concentrations of triglycerides, glucose, and pyruvate in HBL tissue samples indicate lipid consumption as an energy source for tumorigenesis and enhanced glycolysis. Alteration of lactate/pyruvate and glutamine/glutamate concentrations certainly revealed their potential role as diagnostic markers of HBL. In the comparison of HBL samples taken from the center and the border of the tumor, opposite results in terms of these metabolites’ concentrations indicate the importance of these metabolites in diagnostics. From the perspective of this research, we can hypothesize that HBL metastases are favored by enhanced glycolysis and glutamine metabolism; however, further metabolomics studies should be focused on a larger number of samples to explore tissue metabolism from aggressive HBL.

## Figures and Tables

**Table 1:** Chemical shifts, spectral peaks’ multiplicities, and coupling constants of the 16 most important biomarkers in HBLs.

Metabolites		Chemical Shifts (ppm), Peak Multiplicities, and Coupling Constants
Lipids (-CH_3_)	1a	0.83 m
Lipids (-CH_2_-)	1b	1.28 m
Ile (Isoleucine)	2	0.93 t (*J* = 7.4 Hz); 1.00 d (*J* = 7 Hz); 1.25 m; 1.46 m; 1.97 m; 3.67 d (*J* = 3.97 Hz)
Leu (Leucine)	3	0.95 t (*J* = 8 Hz; 9 Hz); 1.70 m; 3.72 m
Val (Valine)	4	0.98 d (*J* = 8 Hz); 1.03 (*J* = 7 Hz); 2.26 m; 3.60 d (*J* = 4 Hz)
Lactate	5	1.33 d (*J* = 7 Hz); 4.10 q (*J* = 7 Hz)
Ala (Alanine)	6	1.46 d; 3.77 q
Pro (Proline)	7	2.00 m; 2.07 m; 2.35 m; 3.34 m; 3.42 m; 4.13 m
Glu (Glutamate)	8	2.04 m; 2.12 m; 2.34 m; 3.75 dd (*J* = 7.19, 4.72 Hz)
Gln (Glutamine)	9	2.13 m; 2.45 m; 3.77 t (*J* = 6.18 Hz)
His (Histidine)	10	3.16 dd (*J* = 7.75 Hz); 3.23 (*J* = 4.93 Hz); 3.98 (*J* = 4.98 Hz); 7.10 d (*J* = 5 Hz); 7.90 d (*J* = 2 Hz)
Cho (Choline)	11	3.19 s; 3.51 dd (*J* = 5.816 Hz, 4.162 Hz); 4.05 ddd
PL (Glycerophosphocholine)	12	3.20 s; 3.62 m; 3.90 m; 4.30 m
Glc (Glucose)	13	3.23 dd (*J* = 9.41 Hz, 7.98 Hz); 3.40 m; 3.46 m; 3.52 dd (*J* = 9.82 Hz, 3.77 Hz); 3.73 m; 3.82 m; 3.88 dd (*J* = 12.30 Hz, 2.23 Hz); 4.63 d (*J* = 7.98 Hz); 5.22 d (*J* = 3.80 Hz)
Tyr (Tyrosine)	14	3.03 dd (*J* = 14.55 Hz, 8.01 Hz); 3.34 dd (*J* = 14.53 Hz, 4.68 Hz); 4.04 dd (*J* = 8.03 Hz, 4.68 Hz); 6.94 m; 7.20 dd (*J* = 7.95 Hz, 1.51 Hz); 7.24 td (*J* = 7.76 Hz, 1.71 Hz)
Phe (Phenylalanine)	15	3.19 m; 3.98 dd (*J* = 7.88, 5.31 Hz); 7.32 d (*J* = 6.96 Hz); 7.34 m; 7.42 m
Formate	16	8.40 s

## Data Availability

The authors confirm that the data supporting the findings are included in the article and the set of raw data that support the reported findings is available from the corresponding author upon request.
